# Predictors of Outcome in Traumatic Brain Injury: New Insight Using Receiver Operating Curve Indices and Bayesian Network Analysis

**DOI:** 10.1371/journal.pone.0158762

**Published:** 2016-07-07

**Authors:** Zsolt Zador, Matthew Sperrin, Andrew T. King

**Affiliations:** 1 Department of Neurosurgery, Salford Royal Foundation Trust, Salford, Greater Manchester, United Kingdom; 2 Institute of Cardiovascular Sciences, Centre for Vascular and Stroke Research, University of Manchester, Manchester, United Kingdom; 3 Health eResearch Centre, Farr Institute, Manchester Academic Health Science Centre, University of Manchester, Manchester, United Kingdom; St Michael's Hospital, University of Toronto, CANADA

## Abstract

**Background:**

Traumatic brain injury remains a global health problem. Understanding the relative importance of outcome predictors helps optimize our treatment strategies by informing assessment protocols, clinical decisions and trial designs. In this study we establish importance ranking for outcome predictors based on receiver operating indices to identify key predictors of outcome and create simple predictive models. We then explore the associations between key outcome predictors using Bayesian networks to gain further insight into predictor importance.

**Methods:**

We analyzed the corticosteroid randomization after significant head injury (CRASH) trial database of 10008 patients and included patients for whom demographics, injury characteristics, computer tomography (CT) findings and Glasgow Outcome Scale (GCS) were recorded (total of 13 predictors, which would be available to clinicians within a few hours following the injury in 6945 patients). Predictions of clinical outcome (death or severe disability at 6 months) were performed using logistic regression models with 5-fold cross validation. Predictive performance was measured using standardized partial area (pAUC) under the receiver operating curve (ROC) and we used Delong test for comparisons. Variable importance ranking was based on pAUC targeted at specificity (pAUC_SP_) and sensitivity (pAUC_SE_) intervals of 90–100%. Probabilistic associations were depicted using Bayesian networks.

**Results:**

Complete AUC analysis showed very good predictive power (AUC = 0.8237, 95% CI: 0.8138–0.8336) for the complete model. Specificity focused importance ranking highlighted age, pupillary, motor responses, obliteration of basal cisterns/3rd ventricle and midline shift. Interestingly when targeting model sensitivity, the highest-ranking variables were age, severe extracranial injury, verbal response, hematoma on CT and motor response. Simplified models, which included only these key predictors, had similar performance (pAUC_SP_ = 0.6523, 95% CI: 0.6402–0.6641 and pAUC_SE_ = 0.6332, 95% CI: 0.62–0.6477) compared to the complete models (pAUC_SP_ = 0.6664, 95% CI: 0.6543–0.679, pAUC_SE_ = 0.6436, 95% CI: 0.6289–0.6585, de Long p value 0.1165 and 0.3448 respectively). Bayesian networks showed the predictors that did not feature in the simplified models were associated with those that did.

**Conclusion:**

We demonstrate that importance based variable selection allows simplified predictive models to be created while maintaining prediction accuracy. Variable selection targeting specificity confirmed key components of clinical assessment in TBI whereas sensitivity based ranking suggested extracranial injury as one of the important predictors. These results help refine our approach to head injury assessment, decision-making and outcome prediction targeted at model sensitivity and specificity. Bayesian networks proved to be a comprehensive tool for depicting probabilistic associations for key predictors giving insight into why the simplified model has maintained accuracy.

## Introduction

Traumatic brain injury remains a global health problem with an approximate incidence of 0.2–0.5% each year [1]. There has been increasing interest in model-based predictions for clinical outcome to improve management strategies, inform patient/relative expectations and also facilitate future clinical trial design [2, 3, 4]. Studies using the International Mission on Prognosis and Analysis of Clinical Trials in TBI (IMPACT) datasets has yielded importance ranking of admission variables. Results highlighted patient age, Glasgow Coma Scale motor score, pupil response and computer tomography (CT) characteristics (Marshall grading) as some of the most influential predictors of clinical outcome [3]. Combination of the datasets from clinical trials in traumatic brain injury has led to score based prediction models [4]. However, statistical techniques in biomedical sciences allow further insight into data prediction. In this study we analyze a series of predictive models using logistic regression and receiver operating curve characteristics to identify influential (“key”) outcome predictors. We then apply Bayesian networks to assess associations of these key predictors with the remaining variables for further insight.

The receiver operating curve (ROC) is a widely used summary indicator assessing prediction accuracy of a binary classification model. It plots sensitivity (true positive fraction) against 1-specificity (true negative fraction), and the area under the curve (AUC) gives a measure of prediction accuracy. AUC, however, considers the entire curve, which is not relevant in every case as regions of interest are often the areas of high sensitivity or specificity. Analysis of standardized partial area under the curve (pAUC) addresses this limitation and allows comparison of predictive performance within a pre-specified region [5,6, 7]. The contribution of distinct variables to prediction accuracy has been assessed based on the increments in pAUC values [6]. In the current study we adopt this approach to establish variable importance ranking based on the decrease in pAUC when the variable of interest is dropped from the predictive model. We then tested if a simplified model can be created using the highest-ranking predictors whilst maintaining equally accurate predictions as the more complex models.

Bayesian networks allow the full depiction of probabilistic relationships between variables [8, 9]. In the biomedical field they have been mainly applied based on their predictive abilities to estimate clinical outcomes for lung [8] and heptocellular cancer [9]. Furthermore automated search algorithms can be applied to build Bayesian networks and with this determine the probabilistic associations between variables. In the second part of the study we used this approach to gain further insight into the data structure and assess how the remaining variables associate with the highest-ranking predictors.

## Methods

### Patient database

We used the open database of the CRASH trial (corticosteroid randomization after significant head injury). This international randomized controlled research collaborative [10, 11] tested the benefit of intravenous corticosteroid infusion following traumatic brain injury. The study included 10008 patients who suffered head injury within 8 hours of initial clinical assessment from 239 hospitals in 49 countries. The CRASH trial database and the full list of variables included in the trial are available online: https://ctu-app.lshtm.ac.uk/freebird/index.php/available-trials/ [12]. We defined admission variables such that they paralleled previous studies by the IMPACT and CRASH Collaborators [2, 3, 4]. The consideration for selecting these variables were that: 1) they reflect the clinical information available to clinicians within a few hours following the injury 2) the clinical relevance of these predictors verified by previously published Nagelkerke R^2^ ranking [3] and 3) these admission variables were available for a substantial number of patients. The variables used in our analysis were: 1) “patient and injury characteristics” which included age, gender, injury cause and severe extracranial injury, the latter defined as an injury which requires hospital admission in its own right [13], 2) “Assessment variables” which consisted of pupillary response, and components of the Glasgow Coma Scale (GCS), the latter being the most widely used universal clinical scale for assessing conscious level (consisting of eye opening, verbal and motor response) [14]. As per trial protocol [11] clinical assessment was carried out within 8 hours of injury. For patients where GCS was not assessable due to intubation the most recent GCS score was recorded. 3) “Imaging findings” which consisted of 1 or more petechial bleed, obliteration of the third ventricle or basal cisterns, subarachnoid bleed, midline shift over 5 mm and intracranial hematoma on computer tomographic scan of the brain. In terms of defining clinical outcome we paralleled previous studies [2, 3, 4] and considered *poor* outcome as death or severe disability and *favorable* outcome as moderate disability or good recovery at 6 months post TBI. Patients for whom all these variables were not recorded were excluded, leaving 6945 patients. Majority of patient excluded using this approach was due to lack of complete brain CT findings for 2191 of the 10008 patients (21,9%) of which 2063 (20,6%) was recorded to not have had a CT brain scan performed at all and only 128 (1,3%) had one or more imaging findings truly missing in the dataset. Full list of 12 predictors, details on variable frequency and missing data are summarized in Table 1. Multiple imputations of missing data were not performed in this study due to the potential technical difficulty of applying our subsequent statistical analysis including Bayesian networks to the imputed data. Furthermore previous analysis of prediction models using the CRASH trial database found similar results for imputed and complete datasets [4]. Past studies on the CRASH datasets also note better outcome at 14 days post injury for high-income countries, compared low-middle income regions, possibly attributable to better infrastructure in the high-income region. However outcomes at 6 months did not show significant difference between the different regions of income [2]. Furthermore the prediction model built on the dataset including data from all levels of incomes were successfully validated with external datasets [4]. Therefore in our study we did not differentiate between data from high and low-middle income regions. The final results of the MRC CRASH trial showed increased morbidity and mortality with administration of methylprednisolonie [15]. However we did not include treatment allocation in our analysis because it would have had no influence on the outcome predictors due the randomization process. This approach is in agreement with the previously published prognostic model based on the CRASH trial database where treatment allocations were also not considered [2]. Furthermore this predictive model was verified using several external databases such as IMPACT or TARN [4, 16] and also single center datasets [17].

**Table 1:** Characteristics of patient data. Summary for frequency distributions of continuous and categorical variables considered in our analysis.

			Complete data only (total 6945 patients)		Missing data only (total 3063 patients)		Entire dataset (total 10008 patients)	
Variable Category	Variable (abbreviation)	Category	Number	Percent	Number	Percent	Number	Percent
Epidemiology	Sex (sex)	male	5706	81.81	2437	79.56	8143	81.36
		female	1239	17.76	626	20.44	1865	18.64
	Age (age)	<20	892	12.79	336	10.97	1228	12.27
		20–24	1191	17.08	501	16.36	1692	16.91
		25–29	860	12.33	442	14.43	1302	13.01
		30–34	754	10.81	367	11.98	1121	11.20
		35–44	1199	17.19	567	18.51	1766	17.65
		45–54	899	12.89	352	11.49	1251	12.50
		>55	1150	16.49	498	16.26	1648	16.47
	Injury Cause (cause)	Road traffic accident	4780	68.53	1638	53.48	6418	64.13
		Fall >2 meters	920	13.19	389	12.70	1309	13.08
		other	1245	17.85	893	29.15	2138	21.36
		no data	NA	NA	143	4.67	143	1.43
	Major extracranial injury (ec)	yes	1638	23.48	559	18.25	2197	21.95
		no	5307	76.09	2266	73.98	7573	75.67
		no data	NA	NA	238	7.77	238	2.38
Assessment	Eye opening (eye)	no response	2680	38.42	688	22.46	3368	33.65
		to pain	1261	18.08	382	12.47	1643	16.42
		to verbal stimulus	1764	25.29	1210	39.50	2974	29.72
		spontaneous	1240	17.78	783	25.56	2023	20.21
	Motor response (motor)	no response	601	8.62	213	6.95	814	8.13
		extension	407	5.84	124	4.05	531	5.31
		abnomal flexion	515	7.38	169	5.52	684	6.83
		withdrawal	933	13.38	303	9.89	1236	12.35
		localises	2723	39.04	1089	35.55	3812	38.09
		follows commands	1766	25.32	1165	38.03	2931	29.29
	Verbal response (verbal)	no response	2640	37.85	645	21.06	3285	32.82
		incomprehensible sounds	1124	16.11	320	10.45	1444	14.43
		single words	821	11.77	402	13.12	1223	12.22
		confused	2006	28.76	1412	46.10	3418	34.15
		orientated	354	5.08	284	9.27	638	6.37
	Pupillary response (pupils)	both reactive	5791	83.03	2266	73.98	8057	80.51
		no response unilateral	496	7.11	92	3.00	588	5.88
		no response	658	9.43	167	5.45	825	8.24
		unable to assess	NA	NA	538	17.56	538	5.38
Imaging findings	Petechial hemorrhage (phm)	yes	1974	28.30	261	8.52	2235	22.33
		no	4971	71.27	612	19.98	5583	55.79
		scan not done	NA	NA	2063	67.35	2063	20.61
		no data	NA	NA	127	4.15	127	1.27
	Subarachnoid bleed (sah)	yes	2206	31.63	255	8.33	2461	24.59
		no	4739	67.94	618	20.18	5357	53.53
		scan not done	NA	NA	2063	67.35	2063	20.61
		no data	NA	NA	127	4.15	127	1.27
	Obliterated 3rd ventricle or basal cisterns (oblt)	yes	1663	23.84	160	5.22	1823	18.22
		no	5282	75.73	712	23.25	5994	59.89
		scan not done	NA	NA	2063	67.35	2063	20.61
		no data	NA	NA	128	4.18	128	1.28
	Midline shift (mdls)	yes	1021	14.64	120	3.92	1141	11.40
		no	5924	84.93	753	24.58	6677	66.72
		scan not done	NA	NA	2063	67.35	2063	20.61
		no data	NA	NA	127	4.15	127	1.27
	Hematoma (hmt)	yes	2718	38.97	362	11.82	3080	30.78
		no	4227	60.60	513	16.75	4740	47.36
		scan not done	NA	NA	2063	67.35	2063	20.61
		no data	NA	NA	125	4.08	125	1.25
Outcome	Outcome at 6 months (outcome)	death or severe disability	2763	39.61	795	25.95	3558	35.55
		Moderate disability or Good recovery	4182	59.96	1815	59.26	5997	59.92
		Alive*	NA	NA	120	3.92	120	1.20
		no data	NA	NA	333	10.87	333	3.33

*Disability data not known

### Predictive variable ranking based on model fit

We applied logistic regression models to predict death or severe disability at 6 months as described above, to parallel the methodology of previous studies [3, 4]. Predictions were done with 5-fold cross validation to avoid over fitting. During this process data were sampled randomly over 6 cycles with each cycle including a 5:1 split of data into training and test datasets. Training datasets were used to fit the prediction model (to “train” the algorithm). This model was then used to predict the variable of interest from the test dataset. To allow comparison to previous studies [3] and a ranking based on “goodness of fit” we used Nagelkerke R^2^ scoring [18]. This technique has been previously used in logistic regression models [3] and it numerically expresses the percentage of variability attributed to a predictor. To allow comparison with these results we first derived importance ranking from the drop in the Nagelkerke R^2^ value for the model produced by excluding the variable of interest as described in previous studies [3].

### Importance ranking based on ROC characteristics

In the first instance we tested a well-established methods of model selection by applying the Akaike Information Criterion (AIC) [19] to backward elimination. This model selection technique penalizes for model complexity against goodness of fit. It starts with the most complex model and after dropping a single variable and reassesses the quality of the model by computing its AIC score at each step. The best model is the one with the lowest AIC score, which represents the best model fit balanced against the complexity of the model. We adopted another approach to model selection where the predictive power of each model was assessed by partial and complete area under the receiver operating curves (pAUC and AUC respectively) and compared using the DeLong’s test [20]. Majority of our analysis focused on areas of high sensitivity and specificity (90–100%) given these are the areas of clinical interest. The entire AUC was also established for comparison. As a new approach to the Nagelkerke R^2^ based ranking, we derived the importance of each predictor from the decrease in standardized pAUC and AUC when the particular predictor was dropped from the model. A greater decrease in these parameters indicated higher importance for the dropped variable.

### Modeling Probabilistic relationships using Bayesian networks

Bayesian networks depict probabilistic relationships between variables using directed acyclic graphs (DAG). The DAG comprises nodes, which in our study represent clinical variables; and edges, which connect nodes indicating the conditional dependence between them. The network can be interrogated for marginal probabilities of a variable, which is the likelihood of the possible categories the variable may take on given the status of the parent node. To establish which network structure best describes the probabilistic relationships between the variables we used the hill-climbing algorithm [21] to search the possible networks. This search process starts with an empirical network structure, then over several iterative steps alters the edges within the network arrive at a structure which best describes the data.

### Model building and predictions

All statistical analysis and model building was carried out in “R” [22], a free software environment for statistical programing and graphics (https://www.r-project.org/). The “bnlearn” [23] package was used for Bayesian Networks analysis. Receiver operating curve analysis was carried using the “pROC” package [7]. Areas under the receiver operating curves were compared using De Long’s test part of the “pROC” package.

## Results

### Variable importance ranking

**Fig 1:**
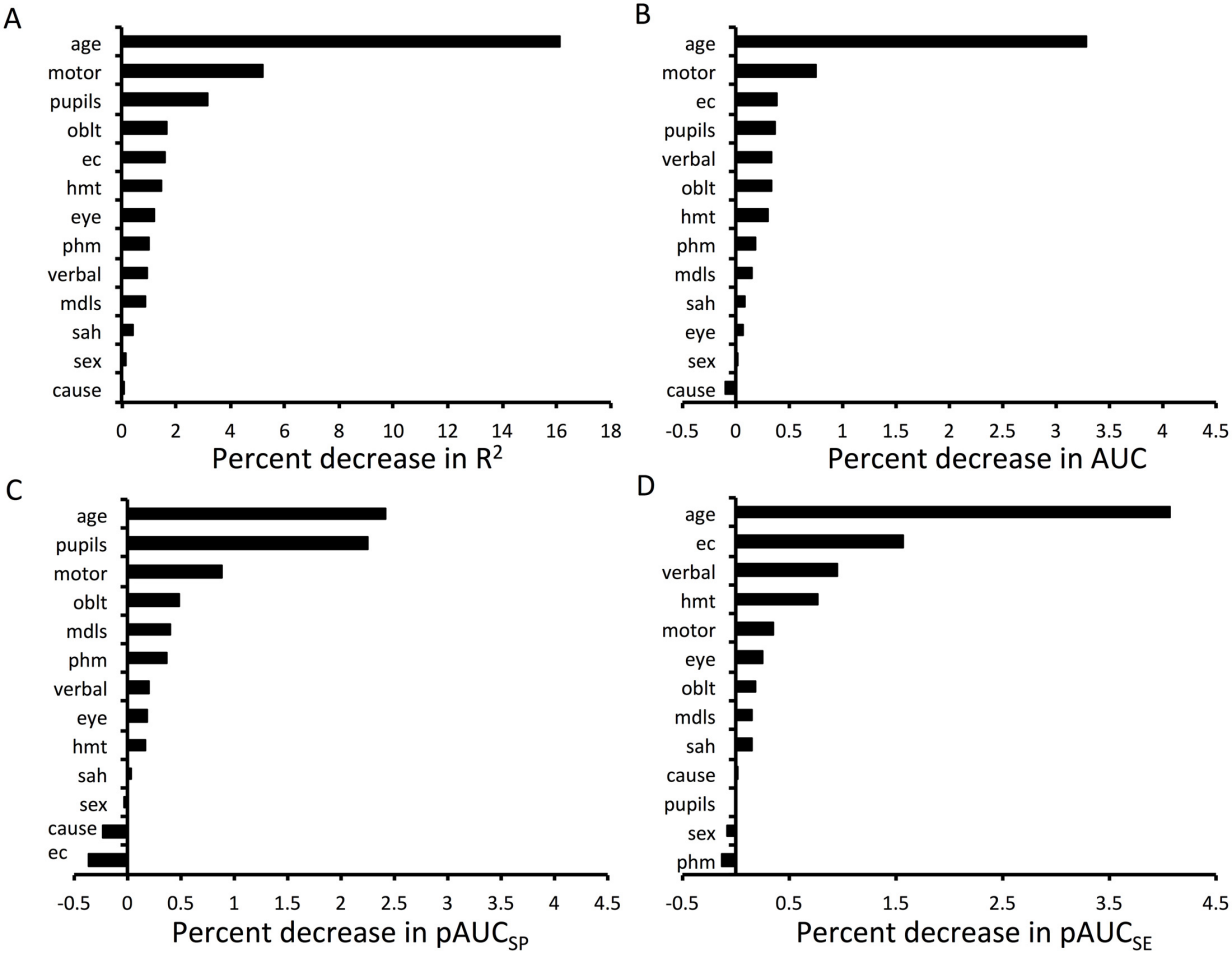
Variable importance ranking. Importance ranking of variables using partial Nagelkerke R^2^ scores (A), ROC characteristics considering the entire AUC (B), pAUC at 90–100% specificity (C) and 90–100% sensitivity (D). See Table 1 for abbreviations.

We categorized admission variables into patient and injury characteristics, assessment or imaging characteristics summarized frequencies in Table 1. Nagelkerke R^2^ ranking confirmed age, GCS motor score, pupillary response, and abnormal CT findings (obliteration of the third ventricle/basal cisterns) as the most influential predictors of poor outcome (severe disability or death) (Fig 1) in keeping with previously published results from the IMPACT dataset [3]. We also ranked admission variables based on their contribution to model predictive power assessed by three different ROC properties (Fig 1):1) the entire ROC curve (AUC based ranking) 2) 90–100% specificity (pAUC_SP_ based ranking) 3) and 90–100% sensitivity range (pAUC_SE_ based ranking). Based on the percent drop in AUC, pAUC_SP_ and pAUC_SE_ values in response to the exclusion the variable of interest we were able to assign an importance ranking, with greater drop translating into a highest rank (also see methods). While the values of percent decrease in pAUC/AUC were small and possibly not meaningful on an absolute scale, it did allow us to make a comparative assessment between the variables and inform subsequent variable selection. The AUC based ranking has confirmed age and motor response as influential predictors of poor outcome (death or severe disability at 6 months post injury) in concordance with the Nagelkerke R^2^ based ranking. Severe extracranial injury also ranked highly followed by pupillary, verbal response and abnormal CT findings. A similar pattern was seen when targeting specificity of 90–100% within the ROC (pAUC_SP_), although extracranial injury was interestingly the lowest ranking variable. However when considering the region of 90–100% sensitivity (pAUC_SE_), age was followed by severe extracranial injury, verbal response, hematoma on CT and motor response. We observed negative values in all AUC based approaches for low ranking variables suggesting that excluding these from the model may improve model accuracy. On potential explanation to this may be an element of overfitting, which occurs in the model including all variables.

### Sensitivity and specificity based model selection

**Fig 2:**
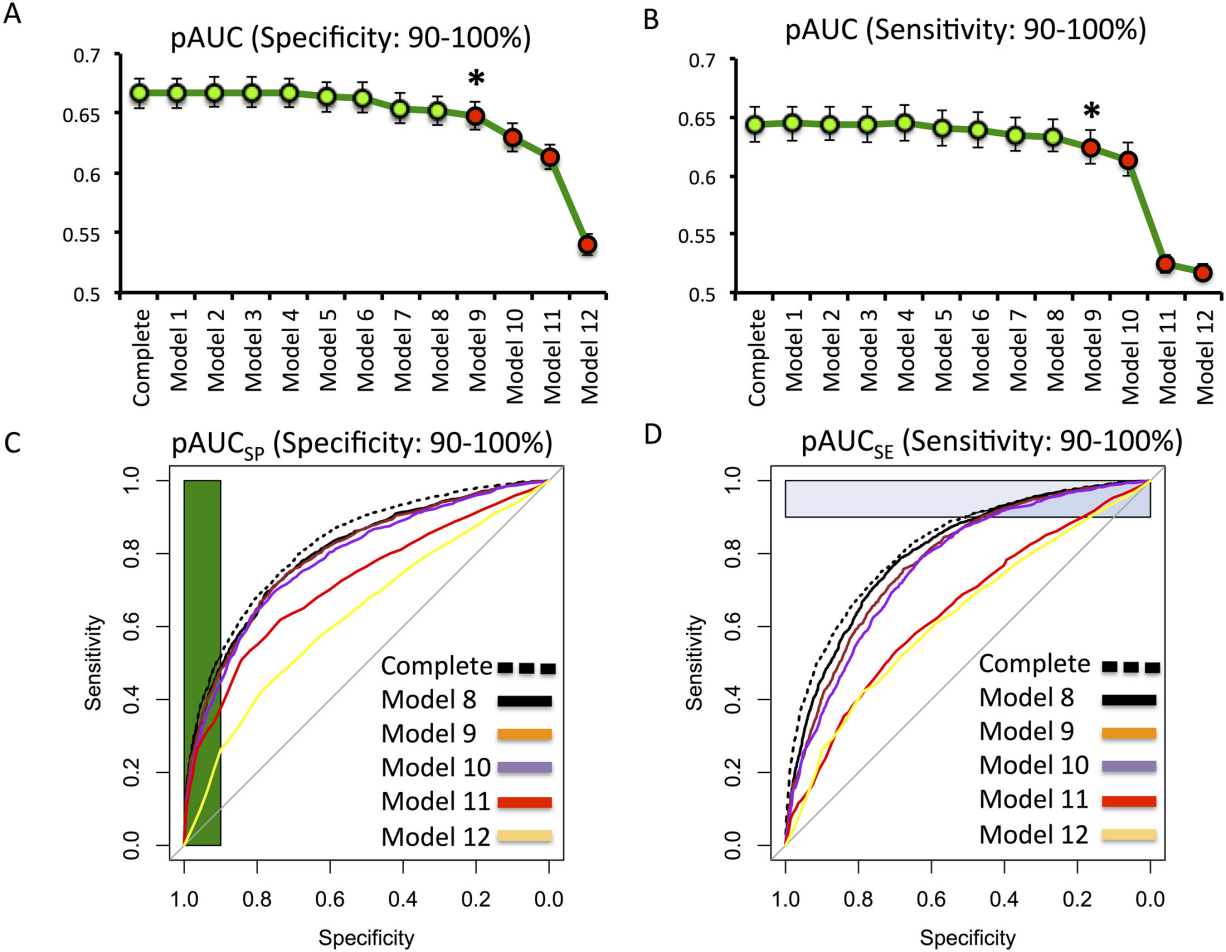
Model selection and comparison of predictive performance. Graph depicting pAUC values for specificity (A) and sensitivity (B) based model selection. See Tables 2 and 3 for model descriptions. Note the progressive decrease in model performance with increasingly simplistic models only becomes significant for model 9 in the specificity based ranking and borderline significance in sensitivity targeted approach. ROC curves of the difference models for the specificity (C) and sensitivity (D) based approach.

We first applied the backward elimination as an established method of model selection to the regression model with all 12 variables included (complete model) to predict severe disability or death at 6 months post TBI. This technique yielded limited simplification of the complete model by suggesting “injury cause” only as a potentially excludable variable (model AIC score 7266.79). We next tested the alternative approach of using area under (AUC) the receiver operating curve (ROC) indices for model selection to create more simplistic models predicting severe disability or death at 6 months. As detailed in the methods with this technique we considered the high specificity (SP) and sensitivity (SE) region of the ROC and incorporates the pAUC ranking described above. For both groups, the lowes ranking variables were excluded in a stepwise fashion until the drop in pAUC compared to the complete model reached significance based on DeLong’s test (Tables 2 and 3). This approach yielded two simplified models (one for specificity and one for sensitivity driven approach) that had the least number of variables but maintained their prediction accuracy compared to the complete model (Tables 2 and 3 and Fig 2). For the specificity driven models the simplified variants pAUC_SP_ was 0.6523 (95% CI: 0.6402–0.6641) only slightly less than the pAUC_SP_ of the complete model: 0.6664 (95% CI: 0.6543–0.679), and this difference was not significant with De Longs test (p = 0.1165). Similarly, with sensitivity driven approach pAUC_SE_ was 0.6332 (95% CI: 0.62–0.6477) compared to the complete models performance of 0.6436 (95% CI: 0.6289–0.6585), which was again not significant with De longs test (p = 0.3448). Comparative analysis of ROC curves are summarized in Fig 2. The variables that were included in these simplified models were labeled as “key predictors” (Tables 2 and 3).

**Table 2:** Summary of model selection using specificity based variable ranking.

Rank	Variable	Model												
		Complete	1	2	3	4	5	6	7	8	9	10	11	12
1	**age**	+	+	+	+	+	+	+	+	+	+	+	+	+
2	**pupils**	+	+	+	+	+	+	+	+	+	+	+	+	
3	**motor**	+	+	+	+	+	+	+	+	+	+	+		
4	**oblt**	+	+	+	+	+	+	+	+	+	+			
5	**mdls**	+	+	+	+	+	+	+	+	+				
6	phm	+	+	+	+	+	+	+	+					
7	verbal	+	+	+	+	+	+	+						
8	eye	+	+	+	+	+	+							
9	hmt	+	+	+	+	+								
10	sah	+	+	+	+									
11	sex	+	+	+										
12	cause	+	+											
13	ec	+												
	pAUC	0.6664	0.6673	0.6678	0.6672	0.6668	0.6635	0.6623	0.6539	0.6523	0.6484	0.63	0.613	0.5401
	CI 95%	0.6543–0.679	0.6544–0.6793	0.6555–0.6802	0.6551–0.6801	0.6552–0.6794	0.6512–0.6758	0.6505–0.6755	0.6416–0.6665	0.6402–0.6641	0.6364–0.6604	0.6181–0.6422	0.6033–0.624	0.5314–0.5491
	DeLong p	NA	0.9191	0.8707	0.9289	0.964	0.7535	0.6474	0.1612	0.1165	0.0439	4.33E-05	1.15E-10	2.20E-16

Stepwise model selection by excluding the least important variable at each step (models 1–12). Variables included in the model are indicated by “+”. Model 1 to 8 maintain their accuracy compared to the complete model (DeLong p values >0.05) with model 8 being the most simplistic. Key variables are (highlighted in bold) are defined as the predictors constituting the most simplistic model. See Table 1 for variable abbreviations.

**Table 3:** Summary of model selection using sensitivity based variable ranking.

Rank	Variable	Model												
		Complete	1	2	3	4	5	6	7	8	9	10	11	12
1	**age**	+	+	+	+	+	+	+	+	+	+	+	+	+
2	**ec**	+	+	+	+	+	+	+	+	+	+	+	+	
3	**verbal**	+	+	+	+	+	+	+	+	+	+	+		
4	**hmt**	+	+	+	+	+	+	+	+	+	+			
5	**motor**	+	+	+	+	+	+	+	+	+				
6	eye	+	+	+	+	+	+	+	+					
7	oblt	+	+	+	+	+	+	+						
8	mdls	+	+	+	+	+	+							
9	sah	+	+	+	+	+								
10	cause	+	+	+	+									
11	pupils	+	+	+										
12	sex	+	+											
13	phm	+												
	pAUC	0.6436	0.6444	0.6442	0.6433	0.6445	0.6408	0.6385	0.635	0.6332	0.6241	0.6138	0.5241	0.5177
	CI 95%	0.6289–0.6585	0.6297–0.6594	0.6304–0.6594	0.6286–0.6587	0.6297–0.66	0.6256–0.6556	0.6241–0.6537	0.6212–0.6495	0.62–0.6477	0.6102–0.6388	0.5999–0.6277	0.5167–0.5323	0.511–0.5242
	DeLong p	NA	0.9382	0.9556	0.9805	0.9323	0.8043	0.6408	0.4234	0.3448	0.07603	0.006258	2.20E-16	2.20E-16

The same principal of model selection was used as for the specificity-based models in Table 2. Key variables are highlighted in bold. See Table 1 for variable abbreviations.

### Probabilistic associations of key predictors with the remaining variables

**Fig 3:**
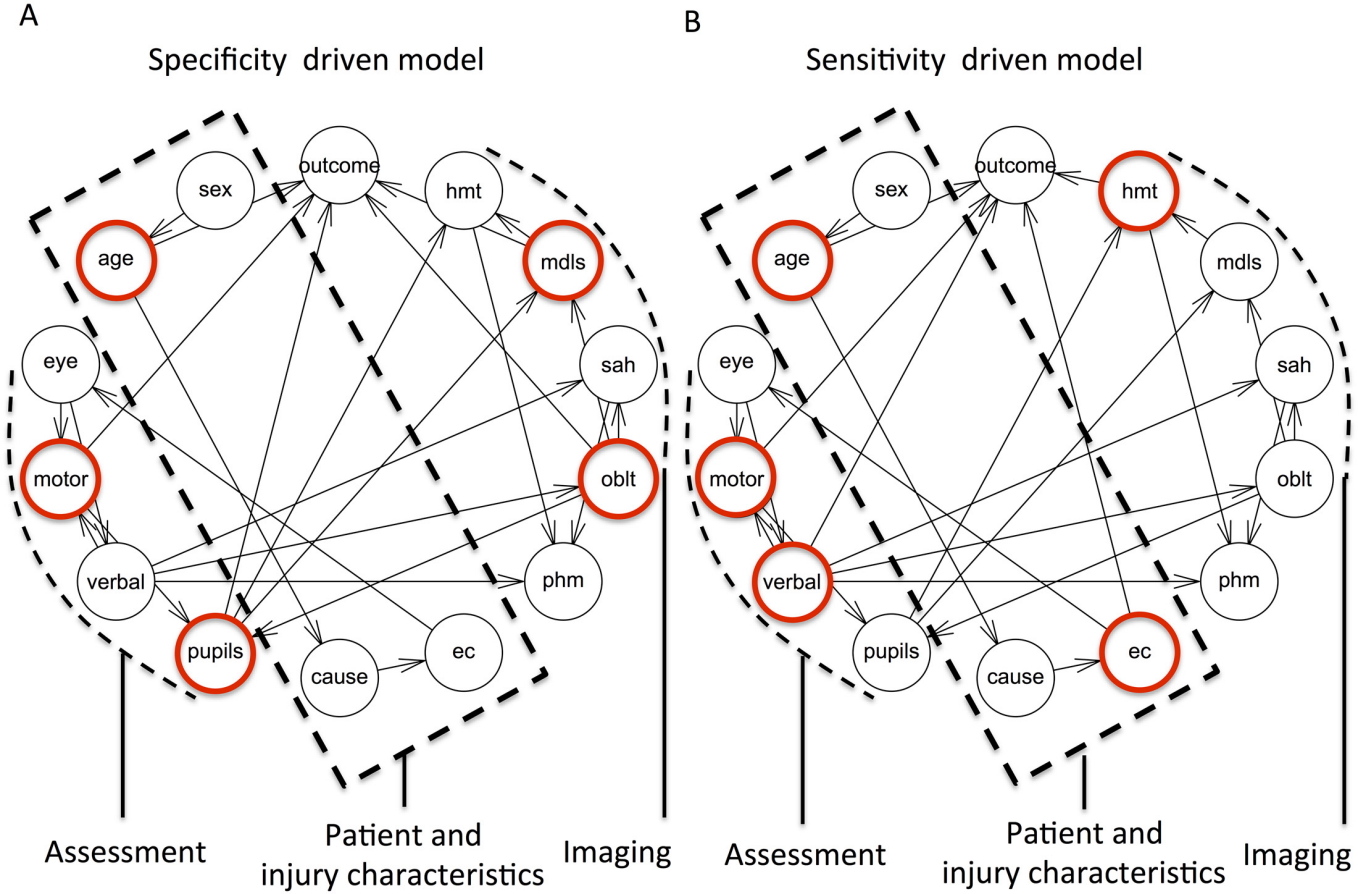
Bayesian network analysis of key predictors. DAG’s depicting probabilistic relationships between “key predictors” (highlighted in red) and the remaining variables. Description in text, see Table 1 for variable abbreviations.

We built Bayesian networks in a partly constrained fashion to analyze the associations of the “key predictors” with other variables (Fig 3). Edges between outcome and the key predictor nodes were pre-fixed and the probabilistic relationships for the remaining variables were explored using the hill-climbing search.

#### Key predictors of the specificity driven model

Age had direct associations with cause of injury and indirectly the presence of extracranial injury. There was a steady increase in the probability of falls with increasing age with 9,4% below age of 20 years compared to 21,7% for age over 55. The likelihood of extracranial injury was 27,2% and 22,2% for RTA and falls respectively compared to 10,4% for other mechanisms (assault, gunshot wound, fall from less than 2 m ect). The probabilistic associations for the remaining variables were mostly multifactorial yielding complex marginal probabilities. Motor response was associated with the remaining components of GCS. Pupillary response was associated with motor response and obliterated third ventricles or basal cisterns on CT. Other interesting observations were that pupillary response was associated with hematoma and midline-shift (both of these are features of mass effect on imaging). On the other hand verbal response was associated with traumatic subarachnoid hemorrhage and petechial bleeds mostly suggesting focal non-mass lesions. Obliteration of the third ventricle/basal cisterns, which can be a consequence of global or focal mass-effect was associated with both verbal and pupillary response.

#### Key predictors of the sensitivity driven model

The associations for age, extracranial injury and verbal response were similar to the specificity driven model. Hematoma on CT was associated with pupil response,midline shift and petechial bleed.

## Discussion

Our study establishes importance measures for outcome predictors in traumatic brain injury using ROC characteristics. We demonstrate the feasibility of this new approach to variable importance ranking based on specificity and sensitivity-based indices. With model selection based on these importance measures we demonstrate that a limited number of the most influential predictors are sufficient to achieve equal predictive performance compared to more complex models. Sensitivity driven analysis highlights the importance of extracranial injury in predicting outcome, which is interestingly not borne out by specificity-based approach. Finally our study is the first to provide insight into the probabilistic associations of these key variables using Bayesian networks.

### Variable importance measures and model selection

Variable importance is relevant in that it helps focus clinical assessment protocols, informs predictive model building and clinical trial design. Conventionally the relevance of outcome predictors was interpreted based on effect sizes and significance levels in uni- or multivariable models [24]. In more recent studies these model outputs were used to inform the weights assigned to variables in predictor score charts [4]. Formal ranking of outcome predictors in traumatic brain injury were first presented by Murray et al [3] which was based on the drop in Nagelkerke R^2^ value, a measure of goodness of model fit applied to the IMPACT database. Our analysis of the CRASH database confirms their findings: age, GCS motor score, pupil response, and abnormal CT findings were the highest-ranking predictors based using the same methodological principals. Subsequent studies assessed increments in AUC values in response to the inclusion of a variable. Important examples are the relevance of considering extracranial injuries [4, 25], CT imaging findings, secondary insults or laboratory parameters in the predictive model [25]. Our analysis of the entire range of AUC showed similar ranking to the Nagelkerke R^2^ based approach except for extracranial injury, which was amongst the more influential variables with entire AUC but not with Nagelkerke R^2^. The conceptual advantage of AUC based ranking used in our study over the Nagelkerke R^2^ is that it reflects the predictive power attributed to each variable rather than “goodness of fit”. On the other hand the drawback of the AUC based ranking is that it considers prediction through the entire curve whereas clinical scenarios are mostly relevant in ranges of high sensitivity and/or specificity. Addressing this shortcoming was the concept of partial AUC introduced by McClish [5]. In their study example the authors demonstrate that including clinical information with cranial CT imaging significantly improves diagnostic accuracy of radiological reporting when considering a single point on the ROC curve. However this benefit is not born out when targeting a range of high specificity of ROC between 90–100%. There were subsequent studies applying this concept of pAUC to demonstrate added predictive power for biomarkers in aneurysmal subarachnoid hemorrhage [6, 7]. We adopted the concept of ranking variables based on the drop in the pAUC values in response to their exclusion from the model, which allowed a sensitivity and specificity driven ranking. The specificity driven ranking was similar mostly to Nagelkerke R^2^ and AUC based ranking, interestingly the sensitivity based ranking showed extracranial injury as an influential predictor. We then “skeletonized” the complete model in a stepwise fashion by excluding the lowest ranking variable. We reassessed changes in predictive power with pAUC values after each exclusion, until it reached significance compared to the complete model containing all the variables. This technique resulted in two simplified models driven by specificity and sensitivity yielding a collection of predictors essential to maintain model accuracy (termed “key predictors” in our study). During this model building process sensitivity or specificity was anchored between 90–100% for sensitivity and specificity based approaches respectively. In practical terms this constraint dictated that at least 9 times out of 10 both models correctly identify patients with *favorable* outcome (moderate disability or good recovery 6 months after TBI) in the specificity-based approach and *poor* outcome (death or severe disability 6 months after TBI) for sensitivity driven models. In terms of clinical translational value, model optimized with the *specificity*-based approach would be useful where the consequences of misclassifying patients into *poor* outcome where the actual outcome is *favorable* (false positive) would have serious consequences. Such scenario is a decision to proceed to a life saving neurosurgical intervention. The trade-off with this approach compared to the *sensitivity*-based model is that a greater number of patients will be misclassified into *favorable* outcome when their actual outcome is *poor*. On the other hand the model optimized for *sensitivity* may be helpful at informing patients patient/relatives expectation regarding poor outcome. Again the trade off for this approach compared to sensitivity-based model selection is that a greater portion of patients who achieve *favorable* outcome will be predicted severe disability or death. Further translational value of the two models is that the “key variables” provide a focus for our clinical assessment during clinical decisions process in the above scenarios.

### Key predictors and their probabilistic associations

We categorized admission variables as patient and injury characteristics, assessment or imaging findings (Fig 3). Our results show that at least one or more variables from each of these categories were amongst the key predictors for sensitivity or specificity driven models. These key predictors were associated with the remaining variables either directly or indirectly in the Bayesian networks. We interpret that through these associations the probabilistic effect of all other variables are carried on to the key predictors supporting their importance. Analysis of these associations revealed some intuitive findings, which mostly overlapped in sensitivity and specificity driven models.

#### Patient and injury characteristics

This section of the network suggests that the demographics of traumatic brain injury appear to be age driven, a key predictor included in both sensitivity and specificity based models. The injury mechanism shifted with older age from road traffic accidents to falls and other mechanisms. Further associations demonstrate the increased likelihood of severe extracranial injury with RTA and falls. Paralleling these findings previous studies showed clinical outcomes worsen with increasing age [26, 27, 28] with low energy falls being four times as common mechanism over the age of 65 [28]. There is emerging relevance of TBI in the elderly population due to its increasing incidence, which is one of the key features of changing epidemiology in traumatic brain injury [1]. There are further suggestions that the plateau observed in the improvement of TBI outcomes since 1990s is partly explained by the worse clinical outcome in the increasing number of elderly patient [29]. Analysis of the Traumatic Coma Data Bank between 1984 and 1987 [30] showed a median age of 25 with 15% of patients over the age of 50. In comparison, another analysis from between 1997–2007 [31] the median age climbed to 45 years for patients suffering TBI with 44% of patients over the age of 50. The factors driving the increased incidence for TBI in the elderly are suggested to be 1) the increasing life expectancy and greater mobility in the elderly [32] 2) preventative measures such as motorcycle helmet laws [33] have successfully reduced the incidence of TBI occurring in traffic accidents which mostly involve younger individuals. The underlying cause for worse clinical outcome in the elderly are multi-factorial and include poor physiological reserve, high incidence of comorbidities [34], use of anticoagulant and anti-platelet medication increasing the risk of intracranial bleeding [1, 28]. While age appears to be a primary outcome prognosticator GCS has historically been used as a triage factor in clinical decision-making. A recently demonstrated implication of age is that older patients appear to present with higher GCS scores compared to the young. This finding prompts us to revisit how the elderly are triaged [28] as higher GCS score is generally associated with better outcome in the general population whereas increasing age dictates worse prognosis as discussed above. Although our network analysis does not show direct association between age and GCS score this is likely explained by the above study analyzing isolated TBI with exclusion of extracranial injuries. With Bayesian networks we were able to formalize the important associations between age, injury characteristics and the remaining variables in a picturesque graph using providing a comprehensive insight into epidemiological properties.

#### Assessment variables

The GCS motor score and pupillary response has been previously highlighted as one of the most influential predictors of clinical outcome [3]. Our specificity driven importance ranking and Bayesian network structure supports this finding. Eye opening and verbal response (the remaining components of the GCS) are both independently associated with motor response, which in turn associates with pupillary response. Motor response was found to be a key predictor in both sensitivity and specificity-based models, whereas verbal response was only deemed influential by the sensitivity driven model. The correlation between the three components of the GCS is intuitive based on the “anatomical sites” of coma, which can be broadly categorized into the supratentorial compartment, bilateral thalamus and brain stem structures (discussed by Bateman [35]). Considering lesions in these locations assessment features of eye opening, verbal and motor response may often be impaired simultaneously and potentially translating into the correlation captured by the Bayesian network in our study. In terms of clinic-radiological correlation components of the GCS may also reflects the character of the intracranial injury. For example abnormal pupillary response can be interpreted as part of compression in brain stem/third cranial nerve, which requires significant focal or global mass effect causing raised intracranial pressure [36]. This is borne out in our network analysis, as pupillary response was associated directly with hematoma, midline shift and obliterated third ventricle on CT. On the other hand verbal response was associated with traumatic subarachnoid hemorrhage and petechial bleeds, lesions that are either not necessarily cause mass effect or more likely to be diffuse in nature respectively. The association between eye opening and severe extracranial injury is not readily explained by clinical intuition and we may speculate it is more of an artifact from facial injuries or pain related discomfort.

#### Imaging features

The specificity driven model highlighted obliterated CSF spaces and midline shift as most influential imaging predictors. Midline shift implies focal mass effect classically due to contusion, focal edema or subdural/extradural hematoma. Previous studies suggested traumatic subarachnoid hemorrhage as an important outcome prognosticator in the context where other imaging features were conveyed by Marshall CT grading [3]. Although we did not find SAH as a high-ranking variable, the network analysis showed it was associated with obliterated CSF spaces and indirectly midline shift and hematoma key predictors of in specificity and sensitivity driven models respectively. Obliterated CSF spaces occur in relation to substantial mass effect and its association with midline shift to supports this intuition. Sensitivity driven model included hematoma as the only influential variable from imaging characteristics. This variable was well connected with the remaining imaging features through midline shift, one of the key indicators of surgical evacuation of hematoma [37]. A limitation of our study is that the cause of the midline shift is not recorded although this feature has a well know impact on clinical outcome.

### Role of extracranial injury in predicting outcome

Prevalence of extracranial injury ranges from 23–41% [2,38] in TBI and the extent of its contribution to clinical outcome has been disputed in the literature. Analysis of the IMPACT database consisting of three observational studies and eight randomized controlled trials in head injury [39] suggested there was some added value from incorporating extracranial injuries when considering the entire patient population [25]. However the importance of extracranial injuries became more pronounced in patients with less severe brain injuries such as high admission GCS or subtle CT abnormalities. A study based on Trauma Research Audit Network (TARN), a prospectively gathered national trauma registry, showed extracranial injury as an important predictor for all head injury severities [40] with greater prognostic effect in less severe TBI. The study noted that exclusion of patients who died within 6 hours of admission reduced the effect of extracranial injury on outcome to a level comparable to IMPACT and CRASH trials. The subgroup of patients who do not survive early stages are unlikely to have been recruited for clinical trials explaining the difference between results. This also highlights the impact of the study population, in particular the recruitment bias of clinical trials making them from this perspective less robust compared to prospective databases. Using the sensitivity-based approach our findings support the prognostic role of extracranial injuries for the entire range of TBI in the CRASH database. One interpretation to this is that TBI severity has a major influence on outcome and severe TBI co-occurs about twice as often with severe extracranial injury than in mild/moderate TBI [40]. However patient with isolated severe head injury (i.e GCS of 8 or below) are also likely to have poor outcome and as a result not always correctly identified for poor prognosis by extracranial injury.

### Study limitations

Our analysis was restricted to variables recorded in the CRASH trial with the simplistic approach of excluding missing data. Previous comparison of prediction models using imputed versus complete data from the CRASH trial showed similar results [4], therefore this may not pose a significant problem from the perspective of this study. Furthermore applying each statistical step of our study including the Bayesian network analysis to multiply imputed data would be technically complex, reaching beyond the scope of this paper.

Majority of the patients were excluded from our analysis due to brain CT scan not being performed. This poses an important limitation to our study and therefore our results should not be generalized to the entire patient population suffering TBI. Another limitation is the element of recruitment bias when using datasets from clinical trials. One example to this is the likely exclusion of early/admission mortality from trial dataset, which as discussed above may offset for example the role of extracranial injuries at influencing clinical outcome. Further limitation is the advances in treatment strategies that have occurred since the trial data was collected (enrollment between 1999 and 2004 for CRASH). External verification of our results using prospectively updated databases such as the Trauma Research and Audit Network could be one potential way to address these shortcomings [41].

## Conclusion

Our study is the first to report sensitivity and specificity based ranking of outcome predictors in traumatic brain injury focusing our clinical assessment on the high-ranking predictors. In a sensitivity driven importance ranking we find extracranial injury as an influential predictor but not with specificity driven models. Bayesian networks provided useful insight into the dependencies between predictors formalizing clinical intuition such as the: 1) age driven aspects of TBI epidemiology 2) radio-clinical correlations between motor, pupillary response with mass effect on CT 3) verbal response and eye opening with non-mass lesions. The application of these techniques to other datasets such as TARN or Collaborative European NeuroTrauma Effectiveness Research in TBI (CENTER-TBI) [42] will provide us with a better understanding of outcome predictors and also hypothesis generation for further studies.
